# Adaptive Cardiac Metabolism Under Chronic Hypoxia: Mechanism and Clinical Implications

**DOI:** 10.3389/fcell.2021.625524

**Published:** 2021-02-02

**Authors:** Zhanhao Su, Yiwei Liu, Hao Zhang

**Affiliations:** ^1^State Key Laboratory of Cardiovascular Disease, National Center for Cardiovascular Diseases, Fuwai Hospital, Chinese Academy of Medical Sciences and Peking Union Medical College, Beijing, China; ^2^Heart center and Shanghai Institute of Pediatric Congenital Heart Disease, Shanghai Children's Medical Center, National Children's Medical Center, Shanghai Jiaotong University School of Medicine, Shanghai, China

**Keywords:** cardiac metabolism, chronic hypoxia, metabolic efficiency, heart function, heart failure, HIF-1α

## Abstract

Chronic hypoxia is an essential component in many cardiac diseases. The heart consumes a substantial amount of energy and it is important to maintain the balance of energy supply and demand when oxygen is limited. Previous studies showed that the heart switches from fatty acid to glucose to maintain metabolic efficiency in the adaptation to chronic hypoxia. However, the underlying mechanism of this adaptive cardiac metabolism remains to be fully characterized. Moreover, how the altered cardiac metabolism affects the heart function in patients with chronic hypoxia has not been discussed in the current literature. In this review, we summarized new findings from animal and human studies to illustrate the mechanism underlying the adaptive cardiac metabolism under chronic hypoxia. Clinical focus is given to certain patients that are subject to the impact of chronic hypoxia, and potential treatment strategies that modulate cardiac metabolism and may improve the heart function in these patients are also summarized.

## Introduction

The human heart is extremely robust in energy metabolism with the highest uptake of oxygen in the body (~0.1 ml O_2_/g/min), and requires ~6 kg of ATP per day, which is 15–20 times its own weight (Kolwicz et al., 2013). To sustain the high energetic demand in the heart, continuous supply of sufficient oxygen is needed. However, a variety of cardiac and pulmonary diseases and systemic pathologies can lead to decreased capacity for oxygen transport and exchange, or decreased blood flow, which reduces oxygen supply to the heart and impairs cardiac energetics.

Hypoxia is the result of the imbalance between oxygen supply and oxygen demand. Under chronic hypoxia, the heart is challenged to produce similar amount of ATP with limited O_2_ for contractile work to maintain normal heart function. This comes in the issue of cardiac metabolic efficiency, which is important in heart function as impaired metabolic efficiency appears to be a prominent feature in heart failure (Bertero and Maack, 2018). In response to hypoxic stimuli, the heart has evolved a delicate adaptive program to maintain metabolic efficiency, in which glucose becomes preferentially used over fatty acid for ATP production. Although the amount of ATP generated from fatty acids is substantially higher than that of glucose (106 vs. 32 molecules of ATP for per molecule of palmitate or glucose respectively), the oxidation of glucose consumes less oxygen compared to fatty acids, which makes it more oxygen efficient (Darvey, 1998). Therefore, when glucose utilization is increased, the coupling of glycolysis and glucose oxidation improves cardiac metabolic efficiency and maintains ATP production (Abdurrachim et al., 2015).

In this review, we will focus on characterizing the major features and the underlying mechanism of adaptive cardiac metabolism under chronic hypoxia from recent studies. The role of altered metabolic adaptation in the progression of cardiac dysfunction in patients with chronic hypoxia and potential available treatment strategies are also discussed.

### Chronic Hypoxia as a Pathophysiologic Component in Cardiac Diseases

**Figure 1:**
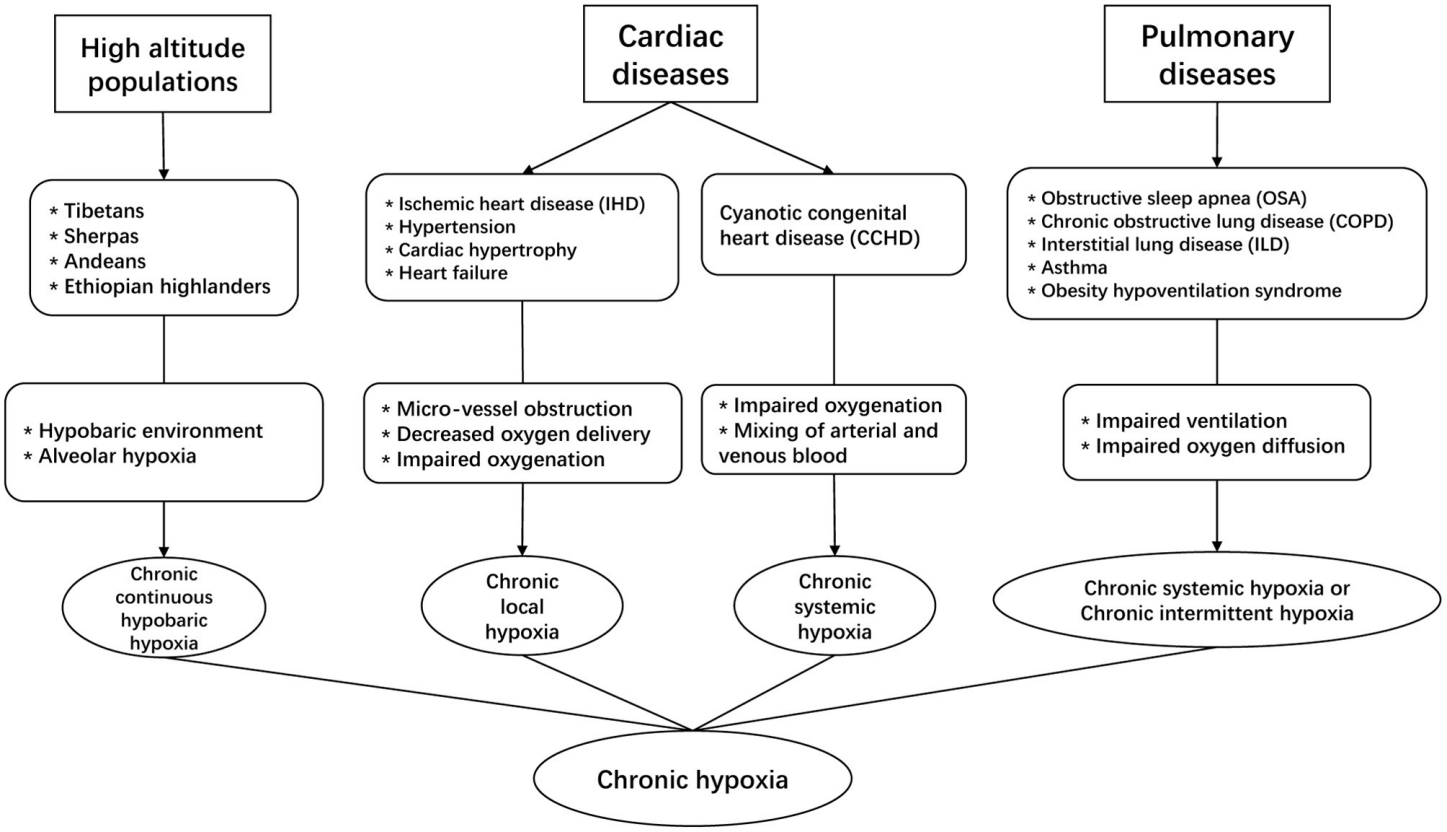
The involvement of chronic hypoxia in different clinical scenarios.

Exposure to chronic hypoxia is a notable feature in high-altitude populations, which constitute ~7% of the world's population, and their number has been rising considerably by 20% since 1990 (Woolcott et al., 2015). These populations are living in a hypobaric and hypoxic environment, which reduce alveolar oxygen level and subject them to the impact of chronic hypobaric hypoxia (Figure 1). From the epidemiologic perspective, hypoxia has major significance as an important risk factor for population health because it is not only the prominent feature in the high-altitude populations, but also involved in the pathophysiology of many cardiac diseases, including ischemic heart disease (IHD), cardiac hypertrophy, hypertension, and heart failure (Abe et al., 2017). In 2019, the prevalent cases of ischemic and hypertensive heart disease were estimated to be 197 million and 18.6 million worldwide, causing a total of 9.14 and 1.16 million deaths respectively (Roth et al., 2020). In these patients, microvascular dysfunction is common and the heart is associated with varying degrees of microvascular obstruction, impaired vascularization and decreased oxygen supply to cardiomyocytes, which causes chronic local hypoxia. In addition, certain congenital heart defects can subject patients to the effect of chronic hypoxia, a condition called cyanotic congenital heart disease (CCHD) (Mahle et al., 2009). In 2019, the number of patients living with congenital heart disease was estimated to be 13 million worldwide (Roth et al., 2020), and nearly 20% of incident congenital heart disease cases in the newborns were CCHD (Zhao et al., 2013).

Chronic hypoxia is also a prominent pathophysiologic feature in some commonly seen pulmonary diseases. For instance, in patients with obstructive sleep apnea (OSA), chronic obstructive pulmonary disease (COPD), or interstitial lung disease (ILD) etc. (Kent et al., 2011; Ryan, 2018), impaired ventilation of air or reduced oxygen diffusion capacity can significantly decrease exchange of oxygen in the lung, which reduces arterial oxygen level. In these pulmonary conditions, COPD and ILD can lead to chronic systemic hypoxia in affected patients, whereas OSA is associated with another type of chronic hypoxia that is characterized by recurrent episodes of hypoxia and reoxygenation, i.e., chronic intermittent hypoxia (CIH). Of note, CIH differs from the continuous exposure to chronic systemic hypoxia as seen in high-altitude populations and CCHD patients. Recent epidemiologic studies suggest that about 10% of the general adult population could suffer from COPD or OSA (McNicholas, 2018), and these conditions are associated with substantially higher risk of cardiovascular co-morbidity and mortality.

Chronic hypoxia has mixed effects on cardiovascular health, whether it is beneficial or detrimental depends on the specific pathophysiologic context and patient risk factors (Savla et al., 2018). In this review, we focus on discussing cardiac metabolism under the context of chronic systemic hypoxia.

### Major Features of the Adaptative Cardiac Metabolism Under Chronic Hypoxia

#### Metabolic Switch of Cardiac Fuels

The human heart is a metabolic omnivore capable to consume all types of available fuels. In the healthy adult human heart, over 95% of ATP are produced from fatty acid and glucose. However, metabolic efficiency differs between these two fuels. Fatty acid has the highest ATP yields on an energy per gram basis, but the oxidation of 1 mol of glucose generates 6.3 high-energy phosphate bonds per mole of O_2_, which is 53.7% higher than that of fatty acid (Kessler and Friedman, 1998). In other words, given a certain amount of oxygen, the use of glucose over fatty acid can yield higher quantity of ATP. Therefore, it has been proposed that increased reliance on glucose might improve cardiac metabolic efficiency and maintain ATP production, which is beneficial in the adaptation to chronic hypoxia (Essop, 2007). In humans, the increased reliance on glucose metabolism is a notable feature in high-altitude populations, such as Tibetans, Sherpas in Nepal, and the Andeans, all of whom are subjected to the chronic effect of hypobaric hypoxia. In a study involving these chronically hypoxic populations, positron emission tomographic (PET) and plasma measurements showed enhanced glucose uptake in the myocardium (0.34 vs. 0.20 μmol glucose/g/min) and lower plasma glucose level (4.7 vs. 5.3 μmol/ml) compared to lowlanders, suggesting a true metabolic adaptation against chronic hypoxia in these populations (Holden et al., 1995). Although the authors had not directly measured the myocardial uptake of fatty acid, plasma level of fatty acid was higher in highlanders than lowlanders (0.65 vs. 0.51 μmol/ml), indicating decreased utilization of fatty acid as metabolic fuels in highlanders (Holden et al., 1995).

Increased reliance on glucose is also a notable cardiac metabolic feature in patients with CCHD. Myocardial samples from patients with tetralogy of Fallot (the most common cyanotic heart defect) showed significantly higher lactate concentrations than acyanotic controls (Modi et al., 2004). Recent data on the surgical samples from CCHD patients also found significantly higher levels of glycolytic intermediates, and the measurement of mitochondrial respiration in primary cardiomyocytes isolated from CCHD patients revealed enhanced substrate utilization with glucose (Liu et al., 2020). Collectively, these data suggest that enhanced utilization of glucose as cardiac fuel is a prominent feature in patients with CCHD. Of note, current studies on the metabolic adaptation under chronic hypoxia in humans are mainly of cross-sectional design, whether this adaptive process occurs progressively through multiple stages require further investigations in future longitudinal studies.

Findings regarding the adaptive cardiac metabolism under chronic hypoxia from human studies corroborate with those from animal studies. In rats exposed to normobaric hypoxia (10% O_2_) for 4 weeks, the left ventricle showed increased reliance on pyruvate for energy production, and the maximal mass-specific and mitochondrial-specific respiration as measured by pyruvate increased by 25% (Ferri et al., 2018). The right ventricle showed similar pattern of changes but the difference appeared non-significant between hypoxic and normoxic myocardium (Ferri et al., 2018). In chronically hypoxic rabbits exposed to 10% O_2_ for up to 5 weeks, the hearts exhibited enhanced ventricular function and increased capacity for glucose oxidation compared to normoxic counterparts (Ross-Ascuitto et al., 2004). In a piglet model of chronic hypoxia (exposed to 8% O_2_ for 4 weeks), the hearts showed elevated baseline glycogen storage levels and increased rates of glycolysis, which conferred protection against ischemic stress (Plunkett et al., 1996). Therefore, various animal models have demonstrated that chronically hypoxic hearts showed increased reliance on carbohydrates for energy production. Notably, a recent study using hyperpolarized ^13^C-magnetic resonance spectroscopy to non-invasively measure pyruvate metabolism showed that in rats exposed to 3-week hypoxia, the conversion of pyruvate into lactate was comparable between hypoxic hearts and normoxic controls (Le Page et al., 2019), which suggests unchanged cardiac metabolism of pyruvate. Nonetheless, given that exposure to chronic hypoxia is associated with reduced utilization of fatty acid (Cole et al., 2016; Mansor et al., 2016), the unchanged metabolism of pyruvate as reported by this study indeed suggests a relatively increased reliance on carbohydrates for energy production in chronically hypoxic hearts, which corroborates with the conclusions from previous studies (Ross-Ascuitto et al., 2004; Ferri et al., 2018).

In addition to carbohydrates, other metabolic fuels with higher energetic efficiency than fatty acid, such as ketones, could have important roles in the adaptive cardiac metabolism. Ketone bodies consist of β-hydroxybutyrate (β-OHB) and acetoacetate. The oxidation of ketones is comparable to glucose and pyruvate in terms of oxygen efficiency (Ferrannini et al., 2016), which makes it an equally efficient fuel under metabolically stressed conditions. This property is potentially beneficial for cardiac adaptation during periods of nutrient or oxygen scarcity. In chronically hypoxic rats, the plasma concentration of β-OHB was significantly elevated (Mansor et al., 2016). Moreover, in primary cardiomyocytes, the presence of β-OHB improves excitation-contraction coupling under hypoxic environment, suggesting its potential role in the protective response against hypoxia (Klos et al., 2019). However, the effect of ketones on the adaptive cardiac metabolism has not been fully explored in animals nor investigated in human patients. Whether ketone bodies contribute to the adaptive cardiac metabolism under chronic hypoxia warrants further studies.

#### Adaptive Changes in Cardiac Mitochondria

Cardiac mitochondria comprise approximately one third of the volume of mature cardiomyocytes and they are well-known for the central roles in energy production and the regulation of various important cellular processes (Tian et al., 2019). Chronic hypoxia can induce a series of adaptive structural and functional changes in cardiac mitochondria. Current studies seem to report different findings due to variations in experimental methodologies and the indicators chosen for measurement. To quantify mitochondrial contents, some studies used the measurement of citrate synthase (CS) activity as the indirect biomarker of mitochondrial content (Larsen et al., 2012). In these studies, the exposure of rats to chronic hypoxia resulted in comparable CS activity in both left and right ventricles with normoxic controls (Heather et al., 2012; Horscroft et al., 2015; Ferri et al., 2018), which suggests little changes in mitochondrial content in the chronically hypoxic myocardium. However, other studies report that in rats exposed to 3-week hypoxia, the ratio of mitochondrial DNA to nuclear DNA was reduced in both ventricles (Nouette-Gaulain et al., 2005), which suggests decreased mitochondrial content. Moreover, morphometric analysis of mitochondria showed that the numerical density significantly increased and the mean volume significantly decreased in both ventricles, indicating an increase in the number of smaller mitochondria (Nouette-Gaulain et al., 2005). The increase in mitochondria number is considered favorable under chronic hypoxia because it can facilitate the mitochondrial transport of oxygen by increasing the surface-to-volume ratio of mitochondria (Nouette-Gaulain et al., 2005).

Mitochondrial oxidative capacity can be altered under chronic hypoxia. These functional changes in cardiac mitochondria should be evaluated in the context of the switch of cardiac fuels. Furthermore, due to diversity in the methodology and the specimens (isolated mitochondria, cells, tissues) to measure mitochondrial oxidation, results from different studies should be interpreted based on the specific methodology and specimen types (Horan et al., 2012). Measurement from bundles of myocardial fibers (non-isolated cardiomyocytes) showed that chronic hypoxia causes a reduction in mitochondrial oxidative capacity as measured with palmitate (Nouette-Gaulain et al., 2005, 2011). The left ventricle showed more rapid declines than right ventricle in mitochondrial oxidative capacity as measured with palmitate, whereas the right ventricle remained relatively unchanged until 3 weeks after exposure to hypoxia (Nouette-Gaulain et al., 2005), suggesting delayed alteration in mitochondrial respiratory chain complexes in the right ventricle. In another study using the similar technique, the measurement of myocardial fibers with pyruvate showed greater mass-specific respiration in the left ventricle (Ferri et al., 2018). The above results are consistent with a recent study, in which primary cardiomyocytes isolated from the left ventricle of hypoxic rats showed an increase in mitochondrial oxidation as measured with pyruvate and decreased mitochondrial oxidation as measured with palmitate (Liu et al., 2020). Some studies involve the isolation of cardiac mitochondria and direct measurement of their respiration. Cardiac mitochondria consist of two anatomically and biochemically distinct subtypes, namely subsarcolemmal mitochondria (SSM) and interfibrillar mitochondria (IFM) (Palmer et al., 1977). In rats exposed to 11% O_2_ for 2 weeks, the hypoxic SSM and IFM showed decreased respiration as measured with fatty acid and pyruvate (Heather et al., 2012). These changes were associated with downregulation of complexes I, II and IV of electron transport chain (ETC) in hypoxic SSM and downregulation of TCA cycle enzyme aconitase in IFM, which indicates decreased functional mass within a mitochondrion (Heather et al., 2012). Taken together, current evidence suggests that at the cellular and tissue level, mitochondrial oxidation capacity appeared to increase for pyruvate and decrease for fatty acids, but in the isolated mitochondria, the oxidative capacity appeared to decrease for both fuels. As we discussed earlier, this may be due to smaller size of mitochondria in chronically hypoxic hearts (Nouette-Gaulain et al., 2005).

The adaptive changes in ETC components are important in hypoxic cardiomyocytes. The mitochondria use oxygen to generate ATP, which can produce reactive oxygen species (ROS) during this process (Giordano, 2005). This issue becomes more prominent under hypoxia, as impaired transport of electron to oxygen and mismatch between electron and oxygen could aggravate ROS production (Guzy and Schumacker, 2006). The downregulation of ETC components in hypoxic SSM was accompanied by reductions in ROS production and offered protection against mitochondrial permeability transition pore (MPTP) opening (Heather et al., 2012), which is beneficial for cell survival under chronic hypoxia. Moreover, the uncoupling protein 3 (UCP3) located in the inner membrane of mitochondria was also decreased by chronic hypoxia (Cole et al., 2016), an adaptive change that decreased proton leak and thereby increased mitochondrial efficiency to use oxygen.

The biosynthesis of another important molecule along the ETC, Coenzyme Q10 (CoQ10), can also be affected under chronic hypoxia. CoQ10 is a vitamin-like organic component of ETC and is ubiquitously expressed in different tissues (Zozina et al., 2018). CoQ10 serves as an important electron carrier in the mitochondrial oxidation and also acts as a potent antioxidant to offset the damaging effect from ROS (Zozina et al., 2018). Given its various biological effect, the importance of CoQ10 should not be neglected. In rats exposed to chronic hypoxia for 3 weeks, reduced cardiac expression of cold-inducible RNA binding protein (CIRBP) caused decreased stabilization of the mRNA responsible for the biosynthesis of CoQ10, which could be associated with reduced ATP production, increased cellular apoptosis, and impaired heart function upon acute stress (Liu et al., 2019). These new findings suggest that under certain pathophysiologic conditions, chronically hypoxic hearts could suffer from impaired protective response against external stress due to its unique remodeling changes within the mitochondria.

#### Adaptive Changes in the High Energy Phosphate System

Chronic hypoxia can induce beneficial adaptive changes in the high energy phosphate system, which is composed of phosphocreatine (PCr) and creatine kinase (CK). This energy buffer system catalyzes the reversible reaction: Phosphocreatine + ADP + H^+^ ↔ Creatine + ATP, and it not only serves as an important shuttle for ATP between mitochondria and muscle fibers, but also a buffer between ATP and ADP levels in the cytosol (Guimaraes-Ferreira, 2014). The ratio of phosphocreatine and ATP (PCr/ATP), as measured by ^31^P Magnetic Resonance Spectroscopy, reflects the energy status of the myocardium. Lowered PCr/ATP ratio is commonly seen in certain cardiac pathological conditions, such as ischemia and heart failure, and it can also be found under hypoxic condition when the heart preferentially uses glucose for metabolism. In the left ventricle, exposure to chronic hypoxia lowers total CK activity, more specifically for the mitochondrial CK isoform (Novel-Chate et al., 1998). In mice exposed to 3-week chronic hypoxia, the heart had decreased PCr/ATP ratio but higher response of ATP/PCr production to PCr changes (Calmettes et al., 2008; Cole et al., 2016), which indicates improved regulation of the balance between energy demand and energy supply. This beneficial adaptation enables the heart to maintain ATP level to withstand the impact of acute hypoxia (Calmettes et al., 2010). As such, the adaptive change of high energy phosphate system from the exposure to chronic hypoxia confers more metabolic resilience for the heart to tolerate ischemic insult.

In human patients, changes in the high energy phosphate system appeared to be consistent with those from animal studies. In a study involving the *in vivo* measurement of phosphocreatine and ATP in high-land Sherpas (Hochachka et al., 1996), cardiac PCr/ATP ratio was shown about half of that in these individuals compared to low-landers, and this difference remained unchanged even after 1-month de-acclimatization from high-altitude environment. These findings suggest the metabolic remodeling toward higher preference for glucose metabolism and increased glucose contribution to aerobic ATP turnover rates in the Sherpa heart (Hochachka et al., 1996). Interestingly, another study measuring cardiac energetics in patients with CCHD reported a PCr/ATP ratio comparable to controls, except that patients with heart failure had significantly lowered PCr/ATP ratio (Miall-Allen et al., 1996). In another groups of patients with Eisenmenger syndrome, which is associated with chronic hypoxemia, PCr/ATP ratio was reported to be significantly lowered than that in normoxic controls (Bowater et al., 2016). These data indicate that in patients with chronically hypoxic hearts, lowered PCr/ATP ratio could be accompanied by the presence of impaired heart function.

### The Mechanism of Adaptive Cardiac Metabolism Under Chronic Hypoxia

#### The Regulatory Roles of Hif-1α and PKC Proteins

**Figure 2:**
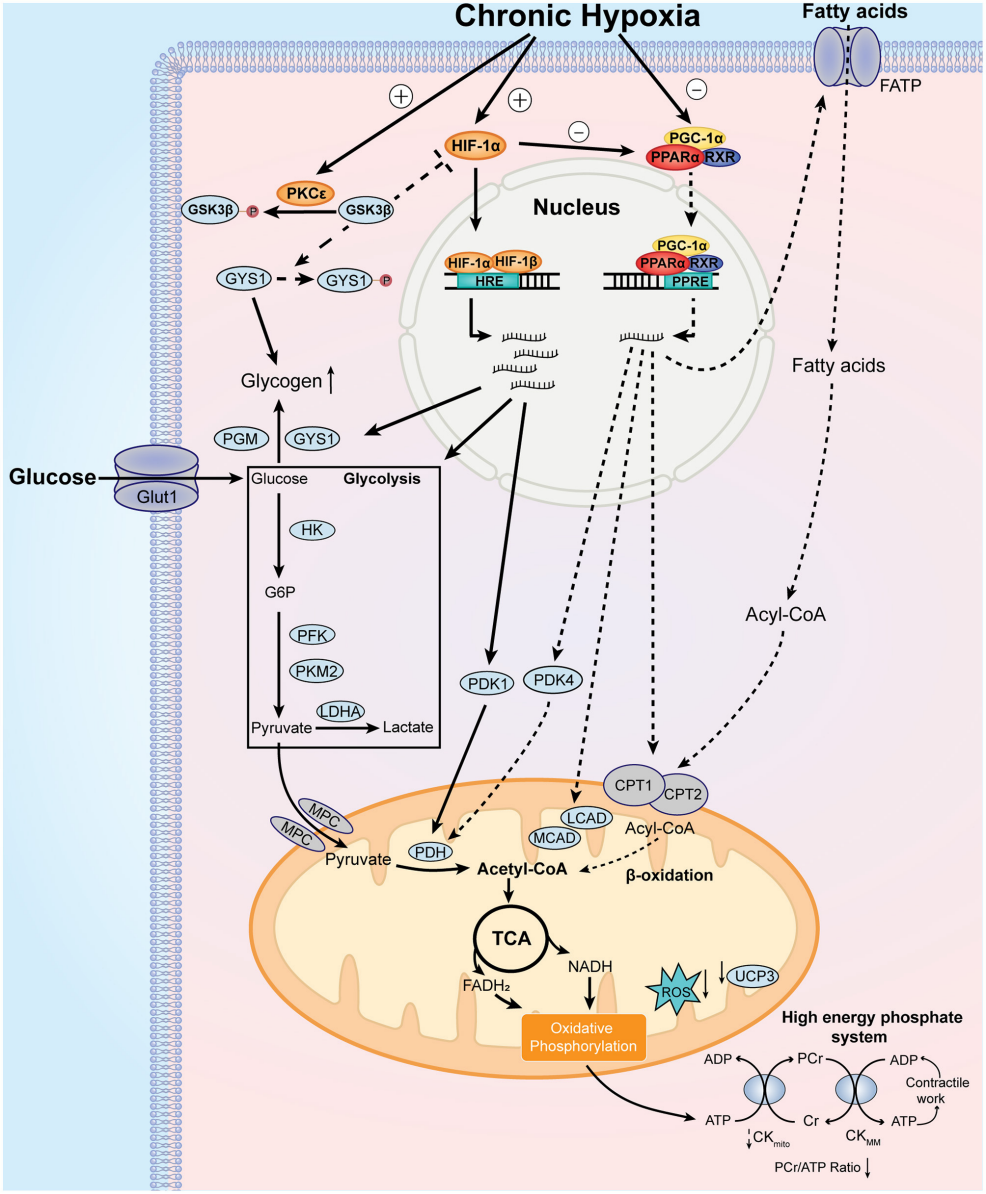
Major features and mechanistic basis of the adaptive cardiac metabolism under chronic hypoxia. Chronic hypoxia leads to increased level and stabilization of HIF-1α, which upregulates the expression of glucose transporter 1 (GLUT1), hexokinase (HK), phosphofructokinase (PFK), pyruvate kinase isozyme M2 (PKM2), lactate dehydrogenase A (LDHA), pyruvate dehydrogenase kinase 1 (PDK1), glycogen synthase 1 (GYS1), and phosphoglucomutase (PGM). These changes lead to increase in glucose uptake, glycolysis, and glycogen synthesis. Additionally, chronic hypoxia decreases PPARα and PGC-1α levels, which decrease fatty acid uptake and β-oxidation through downregulation of fatty acid transport protein (FATP), carnitine palmitoyl transferase 1 (CPT1), medium chain acyl-CoA dehydrogenase (MCAD), long chain acyl-CoA dehydrogenase (LCAD) and uncoupling protein 3 (UCP3). Decreased UCP3 level is associated with less proton leak and increased mitochondrial efficiency for oxygen utilization. Of note, pyruvate dehydrogenase kinase 4 (PDK4) is reduced by downregulated PPARα, which activates pyruvate dehydrogenase (PDH) activity. Decreased PGC-1α reduces mitochondrial biosynthesis of components of electron transport chain, which reduces the generation of reactive oxygen species (ROS). Moreover, protein kinase C epsilon (PKCε) is activated under chronic hypoxia, which phosphorylates glycogen synthase kinase 3β (GSK3β) and reduces its phosphorylation on HIF-1α and GYS1. As the phosphorylation of HIF-1α and GYS1 leads to inhibition of their activities, activated PKCε promotes HIF-1α signaling and glycogen storage under chronic hypoxia. Collectively, these metabolic changes result in increased reliance on carbohydrates over fatty acids for ATP production. Other notable metabolic features include smaller size of mitochondria and decreased phosphocreatine (PCr)/ATP ratio. See text for further details. MPC, mitochondrial pyruvate carrier.

Exposure to chronic hypoxia activates the adaptive cardiac metabolic program through the induction of master regulators. In the myocardium, the physiologic response to hypoxia is mediated by the transcription factors from hypoxia-inducible factor (HIF) family. The biochemical properties and the detailed regulation of Hif-1α by oxygen have been summarized in a previous review (Sousa Fialho et al., 2019). Notably, myocardial expression of Hif-1α mRNA and protein was significantly induced in rats exposed to 10% O_2_ for 3 weeks (Jung et al., 2000; Forkel et al., 2004). In patients with cyanotic heart defects, myocardial samples showed elevated HIF-1α protein levels and increased DNA binding activity, which are directly correlated with the degrees of hypoxemia (Qing et al., 2007). The stabilization of HIF-1α protein binds to hypoxia-response element (HRE) and promotes the expression of metabolic genes that facilitate the utilization of glucose and glycolysis, including glucose transporters 1 (GLUT1), hexokinase (HK), phosphofructokinase (PFK), pyruvate dehydrogenase kinase 1 (PDK1), and lactate dehydrogenase (LDHA) (Semenza, 2014). Moreover, increased HIF-1α promotes glycogen synthesis through induction of glycogen synthase 1 (GYS1) (Pescador et al., 2010) and phosphoglucomutase (PGM) (Semenza, 2014) (Figure 2).

The role of protein kinase C (PKC) in the metabolic adaptation to chronic hypoxia should be mentioned. PKC isozymes are serine/threonine kinases that regulates the protective response against ischemic injury in the heart (Singh et al., 2017). In chronically hypoxic patients and animals, PKCε is activated along with p38 MAP kinase and JUN kinase pathways (Rafiee et al., 2002), which confers cardio-protection in the myocardium. Moreover, the activation of PKCε inactivates glycogen synthase kinase 3β (GSK3β) through phosphorylation, which results in increased accumulation of HIF-1α in the chronically hypoxic heart (McCarthy et al., 2011). Activation of PKC also confers preference for glucose metabolism, with higher rates of glycolysis and glucose oxidation, as well as increased storage of glycogen (McCarthy et al., 2011).

#### The Regulators of Fatty Acid and Mitochondrial Metabolism

The activation of HIF-1α signaling pathway is only half the story, because chronic hypoxia is also associated with downregulation of regulators responsible for fatty acid metabolism. During exposure to hypoxia, both peroxisome proliferator-activated receptor-α (PPARα) and peroxisome proliferator-activated receptor-γ coactivator-1α (PGC-1α) are significantly downregulated (Cole et al., 2016; Mansor et al., 2016), which decreases the expression of their target metabolic genes. The PPARα transcriptional regulatory complex is composed of PPARα, retinoid X receptor (RXR) and PGC-1α, which binds to PPAR response element (PPRE) and regulates fatty acid transport and β-oxidation in the heart through targeting the expression of fatty acid transport protein (FATP), carnitine palmitoyl transferase 1 (CPT1), medium-chain acyl-CoA dehydrogenase (MCAD), long-chain acyl-CoA dehydrogenase (LCAD) (Finck and Kelly, 2002). Importantly, the DNA binding activity of PPARα/retinoid X receptor (RXR) could be reduced by elevated HIF-1α level in the hypoxic hearts (Belanger et al., 2007). PPARα can also regulate glucose metabolism through targeting the expression of PDK2 and PDK4 (Cole et al., 2016; Mansor et al., 2016), which inhibits pyruvate dehydrogenase (PDH) activity through phosphorylation and therefore reduces pyruvate flux. In this sense, PPARα is critical for the mechanistic basis of metabolic switch between fatty acid and glucose. Importantly, the role of these metabolic regulators has been confirmed in genetic studies involving high-altitude populations. For instance, the role of PPARα in the metabolic adaptation in humans was confirmed in several genetic studies of Tibetans (Ge et al., 2012) and Sherpas (Horscroft et al., 2017; Kinota et al., 2018). Genetic variants of PPARα in high-altitude inhabitants is associated with lowered capacity for fatty acid oxidation (Murray et al., 2018), indicating that the metabolic adaption under chronic hypoxia has genetic basis that occurred over generations. Beneficial genetic variants in HIF pathway was also shown in these highlanders (Bigham and Lee, 2014).

PGC-1α is a potent transcriptional coactivator of PPARα that controls cardiac mitochondrial biogenesis (Dorn and Kelly, 2015). The cardiac expression of PGC-1α can be differentially affected by oxygen level, and also seems to differ by left and right ventricle. In one study, chronic exposure to 3-week hypoxia in rats significantly downregulates the mRNA and protein levels of PGC-1α in both left and right ventricle (Ramjiawan et al., 2013). However, another study showed that in rats exposed to 4-week hypoxia, the expression of PGC-1α showed no difference in the left ventricle but was significantly lowered in the right ventricle (Ferri et al., 2018); the expression of another PGC-related family protein, PGC-1β, was significantly elevated in the left ventricle with increased maximal mitochondrial-specific respiration as measured with pyruvate and malate (Ferri et al., 2018). However, in the myocardium of right ventricle from patients with tetralogy of Fallot, cardiac expression of PGC-1α was significantly elevated and was positively correlated with the severity of cyanosis (Zhu et al., 2010). Other proteins involved in PGC-1α regulation cascade, including nuclear respiratory factors (NRF-1/NRF-2) and estrogen-related receptors (ERRs), are important transcriptional factors implicated in mitochondrial biogenesis and can be differentially regulated by hypoxia (Hochachka and Lutz, 2001; Cunningham et al., 2015), which also contribute to the altered mitochondrial biosynthesis under chronic hypoxia. Hence, current studies provide inconsistent evidence between animal and human studies with regard to the regulators of mitochondrial biosynthesis in chronically hypoxic hearts. A deeper understanding on this topic should warrant more comprehensive evaluation of different regulators in future studies.

#### The Regulation of PDH Activity Under Chronic Hypoxia

**Figure 3:**
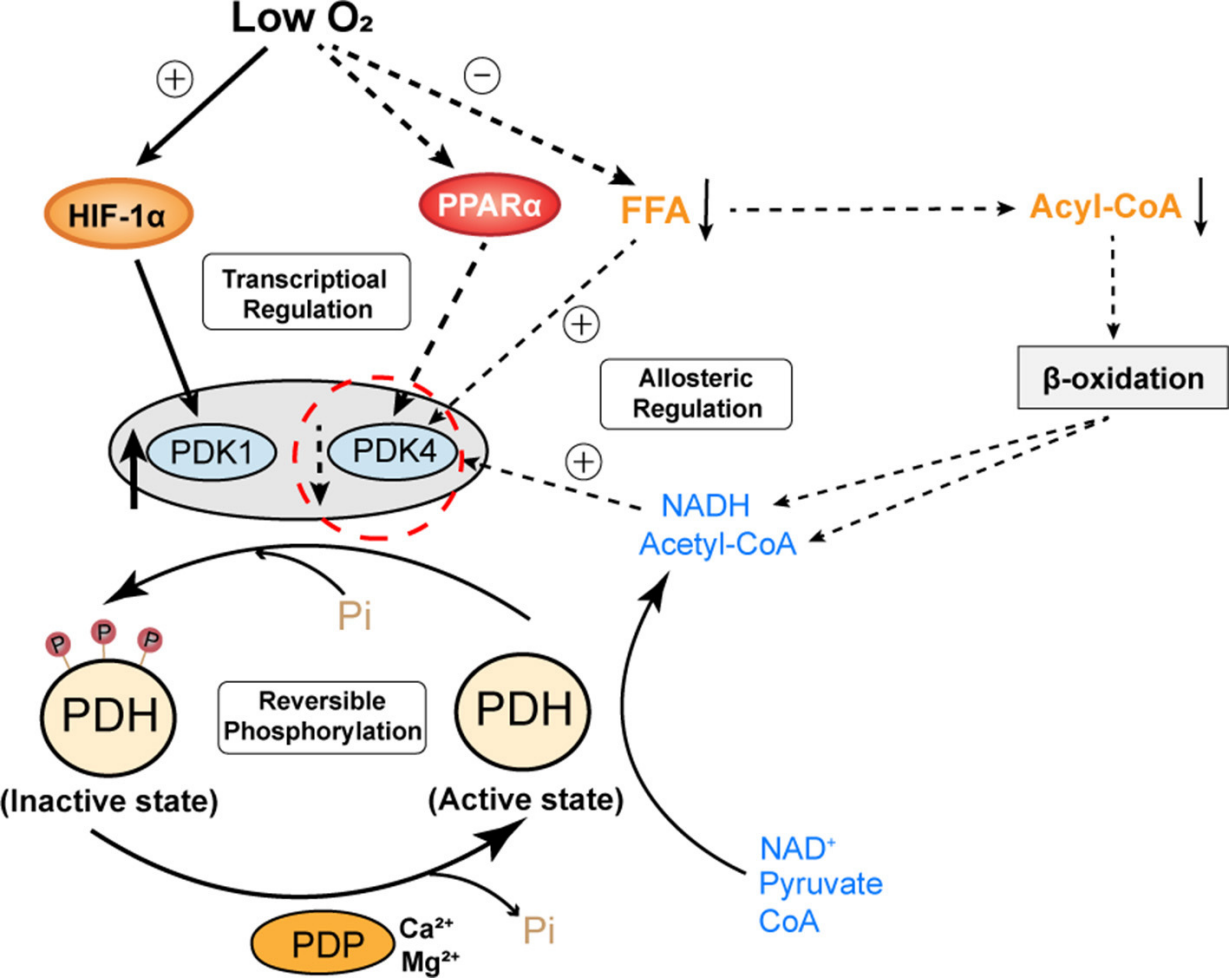
A proposed regulation model of cardiac PDH activity under chronic hypoxia. The activity of PDH is mainly determined by reversible phosphorylation from PDK and PDP. PDK1 is oxygen sensitive and transcriptionally regulated by HIF-1α. PDK4 is nutrient sensitive, its expression is transcriptionally regulated by PPARα and its activity is allosterically regulated by FFA and metabolites (NADH, acetyl-CoA) derived from the oxidation of pyruvate or FFA. Under chronic hypoxia, elevated HIF-1α and decreased PPARα have opposing effects on the expression of PDK1 and PDK4. However, the activity of PDK4 is also affected by decreased levels of fatty acids and metabolites derived from β-oxidation. Hence, PDK4 appears to be more involved in the regulation of PDH activity in the chronically hypoxic heart. Decreased expression and activity of PDK4 reduce the phosphorylation of PDH, which maintains its active state. PDH, pyruvate dehydrogenase; PDK, pyruvate dehydrogenase kinase; PDP, pyruvate dehydrogenase phosphatase; Pi, inorganic phosphate; FFA, free fatty acids; NADH, nicotinamide adenine dinucleotide.

As we mentioned above, the adaptive cardiac metabolism under chronic hypoxia shows increased reliance on carbohydrates for ATP production. The transition from fatty acid to glucose metabolism requires allosteric and transcriptional regulation of key metabolic enzymes that are controlled by HIF-1α, PPARα and PGC-1α. The “Randle cycle” theory proposed in 1960's illustrates the famous ‘glucose-fatty acid cycle’, in which acetyl-CoA generated from fatty acid metabolism inhibits glucose oxidation, or products of glucose metabolism inhibit the entry of fatty acid into mitochondria for β-oxidation (Hue and Taegtmeyer, 2009). This theory forms the basis of adaptive cardiac metabolism. Of note, PDH is the key enzyme that couples glycolysis and glucose oxidation through catalyzing the oxidative decarboxylation of pyruvate, which generates acetyl-CoA that feeds into Krebs cycle (or tricarboxylic acid cycle, TCA cycle). Cardiac PDH activity is regulated by the reversible phosphorylation by PDK and phosphatases (PDP), with phosphorylation resulting in PDH inhibition (Holness and Sugden, 2003). PDK is critical for controlling PDH activity and it can be allosterically regulated by pyruvate, acetyl-CoA and NADH (Holness and Sugden, 2003). Of note, the major PDK isoforms in the heart (PDK1, 2, 4) differ in the kinetic parameters and regulation, such that PDK1 is proposed to be specialized for short-term control of PDH activity, whereas PDK4 is more involved in the regulation of adaptive responses of PDH in circumstances such as starvation (Bowker-Kinley et al., 1998). Moreover, PDK4 appears to be more responsive to intracellular levels of fatty acid, which makes it an important component of the substrate competition via the glucose-fatty acid cycle (Sugden and Holness, 2006). Hence, studies had proposed that PDK4 is an essential component of the cardiac adaptation to maintain the metabolic homeostasis in response to chronic changes in glucose and lipids (Holness and Sugden, 2003). Therefore, in the context of adaptation to chronic hypoxia, PDK1 (as regulated by HIF-1α) and PDK4 (as regulated by PPARα) seems to be more involved in the regulation of PDH activity (Figure 3).

Despite the importance of PDK in the regulation of PDH activity, current studies seemingly reported mixed findings on the relative changes of PDK expression in chronically hypoxic hearts. In the left ventricle, while some studies reported significantly reduced expression of PDK4 (Cole et al., 2016; Mansor et al., 2016), other studies reported unaltered levels of PDK1, 2, and 4 (Heather et al., 2012; Handzlik et al., 2018). An earlier study even reported significantly elevated PDK4 in both left and right ventricles after 14 days of exposure to hypobaric hypoxia (Sharma et al., 2004). Differences in hypoxic models (i.e., length of exposure to hypoxia, the presence of low atmospheric pressure), animal species, and measurement techniques between these studies could be responsible for these mixed results. As we discussed above, chronic hypoxia is associated with elevated myocardial HIF-1α and suppressed PPARα, therefore, it is more plausible that in chronically hypoxic hearts, PDK1 is induced by myocardial HIF-1α, whereas PDK4 level is decreased due to lowered PPARα. In this case, the opposite direction of changes in PDK1 and PDK4 in chronically hypoxic hearts appears to produce opposing regulatory effects on cardiac PDH activity. Importantly, this theory may explain why cardiac PDH activity and pyruvate flux in chronically hypoxic hearts was comparable to that in normoxic controls in some animal studies (Handzlik et al., 2018; Le Page et al., 2019). It could also explain why some studies reported that the oxidation of glucose or pyruvate was increased in chronically hypoxic hearts compared to normoxic hearts (Ross-Ascuitto et al., 2004; Ferri et al., 2018). Given these mixed findings, further studies are warranted to confirm the relative changes in the expression level and activity of PDKs in the chronically hypoxic hearts.

### Maladaptive Cardiac Metabolism and Cardiac Dysfunction Under Chronic Hypoxia

The flexibility in the switch of metabolic fuels underlies cellular adaption to chronic hypoxia. Current evidence suggests that hearts adapted to chronic hypoxia could maintain ATP production and provide sufficient cardiac work. However, this exquisite energy balance could be perturbed by various factors that are associated with reduced glucose utilization, which decreases cardiac metabolic efficiency. Indeed, decreased cardiac metabolic efficiency has been proposed to impair mitochondrial energetics and increase the risk of heart failure in patients with diabetes (Boudina and Abel, 2006). Here, we illustrate certain scenarios in which maladaptive cardiac metabolism could be associated with the development of cardiac dysfunction under chronic hypoxia.

Despite the critical role of HIF-1α in the adaptive response to chronic hypoxia, it is important to note that the regulation of myocardial HIF-1α could be implicated with signaling pathways other than oxygen sensing, such as insulin signaling pathway. Insulin modulates HIF-1α protein synthesis and activity via phosphatidylinositol-3 kinase (PI3-K) and mitogen-activated protein kinase (MAPK) pathways (Doronzo et al., 2006). Furthermore, insulin signaling pathway promotes the stabilization of HIF-1α complex, thereby inducing HIF-1α target genes (Zelzer et al., 1998). In diabetic hearts, the presence of insulin resistance attenuates the expression of HIF-1α protein, which impairs HIF-1α-mediated signaling and adaptation to hypoxia (Dodd et al., 2018). In rats exposed to prolonged hypoxia (10% O_2_), sustained elevation of myocardial Hif-1α regulates the metabolic switch from fatty acid to glucose for ATP production since birth. However, when they enter puberty, the rise of insulin resistance attenuates myocardial expression of Hif-1α and reduces the capacity of the heart to use glucose, which disrupts the adaptive cardiac metabolic program, decreases ATP production and leads to cardiac dysfunction (Liu et al., 2020). As increased insulin resistance could blunt the HIF-1α driven response to hypoxia, this may explain a recent finding that children from Tibet had reduced left ventricular function at pubertal age (Qi et al., 2015). In this high-altitude population, increased insulin resistance during puberty might attenuate myocardial HIF-1α signaling, which impairs the adaptive cardiac metabolism under chronic hypoxia.

Insulin resistance can prevent the heart from using glucose effectively. Therefore, insulin resistance is particularly relevant in the chronically hypoxic populations who have increased reliance on glucose metabolism. The presence of metabolic risk factors, including obesity and hyperglycemia, has been noted to be independently associated with left ventricular diastolic dysfunction in high-altitude populations (Zheng et al., 2019). The development of insulin resistance is also commonly observed in patients with chronic hypoxia, such as obstructive sleep apnea (Ip et al., 2002) and cyanotic congenital heart disease (Niwa, 2019). In these patients, the presence of insulin resistance disrupts the utilization of glucose, which impairs cardiac efficiency and alters the adaptive cardiac metabolism under chronic hypoxia. As a result, these patients could be particularly susceptible to cardiac dysfunction and heart failure when metabolic diseases are present. Therefore, early intervention strategies to control metabolic risk factors could translate into cardiovascular benefits for the chronically hypoxic population.

The long-term outcomes of the adaptive cardiac metabolism in persons exposed to the effect of chronic hypoxia remains to be determined. After all, this is not a “physiological” status for the heart because fatty acid is the major metabolic fuel in the normoxic state. As patients become older, newly-developed co-morbidities or the worsening of pre-existing cardiopulmonary diseases could also pre-dispose to heart failure. Future studies are warranted to investigate and dissect the long-term impact of the adaptive cardiac metabolism on heart function in patients with chronic hypoxia.

### Potential Strategies to Modulate Adaptive Cardiac Metabolism Under Chronic Hypoxia

**Figure 4:**
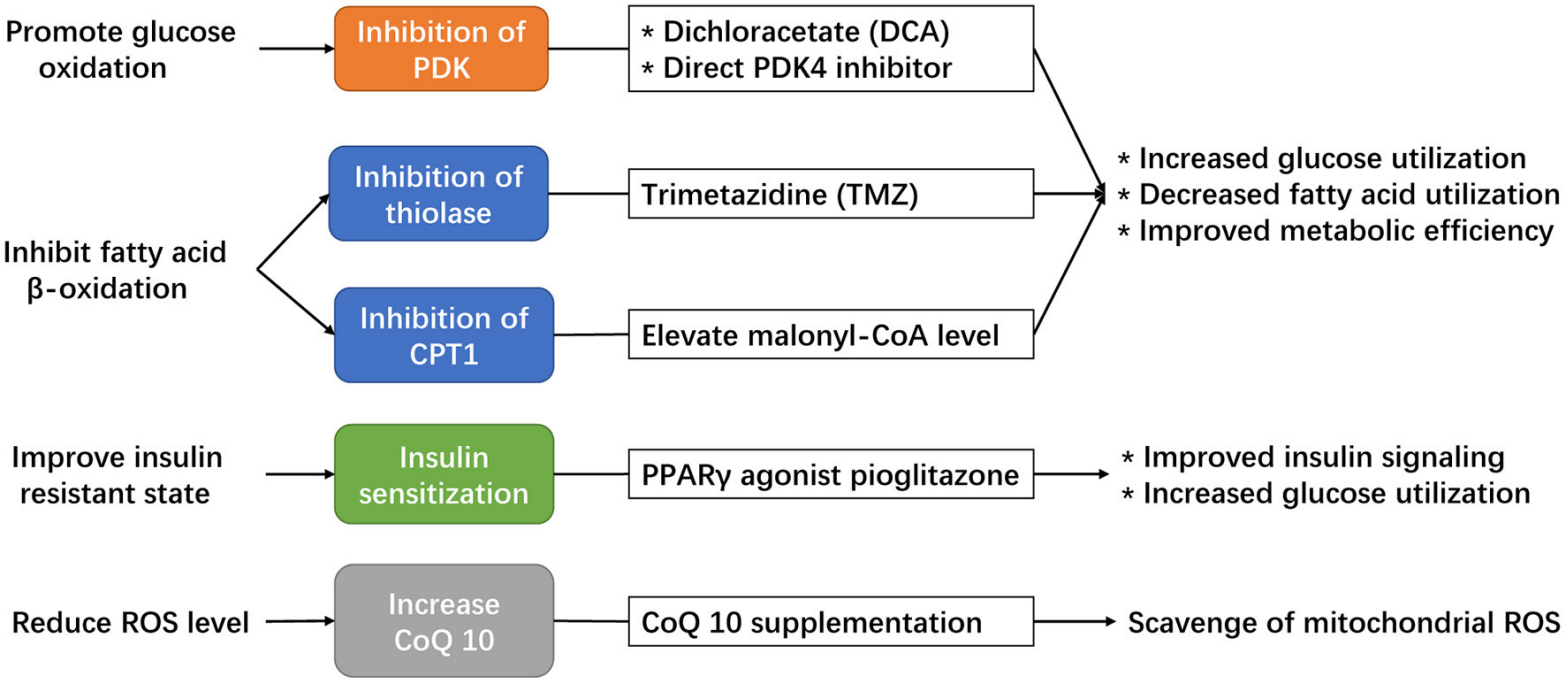
Potential strategies available to modulate the adaptive cardiac metabolism under chronic hypoxia.

As we discussed above, in chronically hypoxic patients, any disruptions in the utilization of glucose, including uptake, degradation, or catabolism, could result in cardiac energy deficit and pre-disposition to cardiac dysfunction. Therefore, potential treatment strategies should increase glucose uptake or target the coupling of glycolysis and glucose oxidation to improve cardiac metabolic efficiency and maintain ATP production for contractile work in this patient population. Currently, the number of potential drug targets is limited. There are several available drugs that can promote glucose metabolism, but none have been tested in patients with chronic hypoxia (Figure 4).

The first is dichloroacetate (DCA), a direct inhibitor of PDK enzyme that serves to increase PDH activity. DCA has been shown to improve the coupling of glucose oxidation to glycolysis in hypertrophied hearts (Lydell et al., 2002), which might offer a potential benefit in chronically hypoxic hearts to promote the formation of adaptive metabolism. Indeed, the use of DCA in rat exposed to chronic hypoxia has already been shown to increase cardiac pyruvate flux, which elevates the stockpile of acetyl-carnitine, maintains ATP production, and improves tolerance to acute hypoxic stress (Handzlik et al., 2018). DCA treatment can also decrease oxidative stress and prevent cell death (Kato et al., 2010), which is beneficial in hypoxic cardiomyocytes. However, the half-life of DCA is short (Kim and Chauhan, 2018) and might hinder its clinical translation.

The second available drug is trimetazidine (TMZ), an established antianginal drug that inhibit fatty acid metabolism through inhibition of mitochondrial 3-ketoacyl thiolase and enhance PDH activity to improve the metabolic efficiency in the myocardium (McCarthy et al., 2016). The administration of TMZ in hypoxic cardiomyocytes promotes glucose oxidation and was protective against hypoxia-induced injury (Wei et al., 2015), however, the effect of TMZ in hypoxic animal models has not been explored. Moreover, clinical studies of TMZ showed mixed results regarding its clinical benefits. In a meta-analysis involving 995 patients with heart failure (Gao et al., 2011), the use of TMZ was associated with significant improvement in left ventricular ejection fraction, end-systolic volume, NYHA classification, as well as protective effect for all-cause mortality, cardiovascular events and hospitalization. However, in a latest clinical trial, the use of TMZ in patients after percutaneous coronary intervention failed to improve the composite endpoint of cardiac death, hospitalization for cardiac events, or angina leading to angiography or changes in antianginal medication (Ferrari et al., 2020). These contradictory results indicate that TMZ might provide clinical benefits in certain patient subgroups, such as those with an established diagnosis of heart failure, but not in other subgroups. Therefore, in patients with chronic hypoxia, those with impaired cardiac function could be the population likely to benefit from using TMZ.

The third option is CPT1 inhibition, which could result in decreased mitochondrial uptake of long-chain fatty acid, reduced β-oxidation, and promotion of glucose oxidation. Although several synthetic CPT1 inhibitors, including oxfenicine, etormoxir and perhexiline, have been developed in the past decades, none of them have made into clinical practice to treat heart failure (Heggermont et al., 2016). Malonyl-CoA is a natural inhibitor of CPT1, and pre-clinical studies had shown that modulation of malonyl-CoA, or more specifically, increasing cardiac malonyl-CoA level or inhibition of malonyl-CoA decarboxylase, can lead to improvement in cardiac function by decreasing fatty acid oxidation and increasing cardiac metabolic efficiency (Fillmore and Lopaschuk, 2014). In this case, indirect inhibition of CPT1 through modulating malonyl-CoA could be a feasible strategy to improve cardiac metabolic efficiency in chronically hypoxic patients.

In recent years, novel strategies are emerging to modulate cardiac metabolism by direct targeting of specific regulators. For instance, direct modulation of cardiac PDK4 activity could offer a potentially feasible strategy to enhance glucose metabolism in chronically hypoxic hearts. A recent study found that conditional cardiac deletion of PDK4 could result in an increase in PDH activity and consequently an increase in glucose relative to fatty-acid oxidation, which was associated with reduced cardiac fibrosis and improved left ventricular function in mice following myocardial infarction (Cardoso et al., 2020). The recent discovery of oral compound that specifically inhibit PDK4 could offer a potential strategy for clinical translation (Lee et al., 2019).

As we had discussed above, insulin resistance and altered insulin signaling could impair the protective HIF-1α pathway in response against chronic hypoxia. Hence, another potentially feasible strategy to maintain the adaptive cardiac metabolism under chronic hypoxia is by improving cardiac insulin sensitivity. Pioglitazone is a potent insulin sensitizer that targets PPARγ signaling to decrease systemic and myocardial insulin resistance (Soccio et al., 2014), with favorable effects on cardiac metabolism (Young, 2003). In animal studies, pioglitazone has been demonstrated to promote myocardial glucose uptake, enhance glucose oxidation, and improve contractile function in isolated hearts of rats with insulin resistance (Golfman et al., 2005). In patients with T2DM, pioglitazone has been shown to improve left ventricular diastolic function and cardiac output (van der Meer et al., 2009; Clarke et al., 2017). A recent study shows that the use of pioglitazone in pubertal hypoxic rats could attenuate insulin resistance and restore the protective cardiac Hif-1α signaling in the hypoxic myocardium, which maintains the adaptive cardiac metabolism and preserves cardiac function (Liu et al., 2020). More animal and human studies should test this strategy in chronically hypoxic patients with increased insulin resistance to preserve adaptive metabolism and reduce the risk of heart failure.

Modulation of CoQ10 biosynthesis, or direct supplementation of CoQ10, should be considered in patients with chronic hypoxia. Although the biological effects of CoQ10 have been reported in various cardiac diseases (Zozina et al., 2018), its biological relevance and clinical benefits are yet to be determined in patients with chronic hypoxia. Given its potent antioxidant effect and involvement in mitochondrial respiration, the addition of CoQ10 in chronically hypoxic patients may improve their adaptive cardiac metabolism and produce clinical benefits. The clinical benefits of CoQ10 has been established in patients with heart failure (Sharma et al., 2016), and it is reasonable to test the effects of CoQ10 on patients with chronic hypoxia in future studies.

## Conclusions

In response to chronic hypoxia, the hearts initiate transcriptional programs to increase reliance on carbohydrates over fatty acid for ATP production, with altered mitochondrial structure and function that improve metabolic efficiency and reduce ROS generation, as well as beneficial changes in glycogen storage and high energy phosphate system. Collectively, the adaptive cardiac program improves the regulation of the balance between energy demand and energy supply when oxygen is limited. Of note, this adaptive program is prone to be disrupted by systemic pathological factors, such as insulin resistance, and it can be complicated with other pathological factors concomitantly seen in patients with chronic hypoxia. The presence of these risk factors can pre-dispose to altered adaptive metabolism and cardiac dysfunction. Currently available pharmacological strategies are limited and none have been tested in patients with chronic hypoxia. Therefore, more investigational studies are needed to unravel the critical features and mechanism for maladaptive cardiac metabolism in these patients.

## Author Contributions

ZS and YL contributed to the conception of the article. ZS drafted the first manuscript and YL contributed to the revision of the manuscript and graphical design. HZ contributed to the critical revision of the manuscript. HZ and YL obtained funds. All authors approved the submission of the article.

## Conflict of Interest

The authors declare that the research was conducted in the absence of any commercial or financial relationships that could be construed as a potential conflict of interest.
